# An Employment Intervention Program (Work2Prevent) for Young Men Who Have Sex With Men and Transgender Youth of Color (Phase 1): Protocol for Determining Essential Intervention Components Using Qualitative Interviews and Focus Groups

**DOI:** 10.2196/16384

**Published:** 2020-08-10

**Authors:** Brandon J Hill, Darnell N Motley, Kris Rosentel, Alicia VandeVusse, Robert Garofalo, John A Schneider, Lisa M Kuhns, Michele D Kipke, Sari Reisner, Betty M Rupp, Maria Sanchez, Micah McCumber, Laura Renshaw, Matthew Shane Loop

**Affiliations:** 1 Planned Parenthood Great Plains Overland Park, KS United States; 2 Center for Interdisciplinary Inquiry and Innovation in Sexual and Reproductive Health Department of Obstetrics and Gynecology University of Chicago Chicago, IL United States; 3 Guttmacher Institute New York City, NY United States; 4 Division of Adolescent Medicine, Ann & Robert H Lurie Children’s Hospital Department of Pediatrics, Feinberg School of Medicine Northwestern University Chicago, IL United States; 5 Department of Medicine University of Chicago Chicago, IL United States; 6 Division of Research on Children, Youth, and Families Children's Hospital Los Angeles Los Angeles, CA United States; 7 Fenway Health The Fenway Institute Boston, MA United States; 8 Collaborative Studies Coordinating Center Department of Biostatistics, Gillings School of Global Public Health University of North Carolina at Chapel Hill Chapel Hill, NC United States

**Keywords:** HIV/AIDS, YMSM, YTW, GNC youth, LGBTQ, unemployment, homelessness, sex work

## Abstract

**Background:**

HIV continues to have a disparate impact on young cisgender men who have sex with men (YMSM), young trans women (YTW), and gender-nonconforming (GNC) youth who are assigned male at birth. Outcomes are generally worse among youth of color. Experiences of discrimination and marginalization often limit educational attainment and may even more directly limit access to gainful employment. Though seemingly distal, these experiences influence young people’s proximity to HIV risk by limiting their access to health care and potentially moving them toward sex work as a means of income as well as increased substance use. Work2Prevent (W2P) aims to achieve economic stability through employment as a structural-level intervention for preventing adolescent and young adult HIV infection. The study will pilot-test an effective, theoretically driven employment program (increased individual income and independence [iFOUR]), for HIV-positive adults, and adapt it to the needs of black and Latinx YMSM, YTW, and GNC youth aged 16 to 24 years who are vulnerable to HIV exposure.

**Objective:**

This paper aimed to describe the protocol for the exploratory phase of W2P. The purpose of this phase was to determine the essential components needed for a structural-level employment intervention aimed at increasing job-seeking self-efficacy and career readiness among black and Latinx YMSM, YTW, and GNC youth aged 16 to 24 years.

**Methods:**

The exploratory phase of the W2P study consisted of in-depth interviews and focus groups with members of the target community as well as brief interviews with lesbian, gay, bisexual, transgender, and queer (LGBTQ)–inclusive employers. The study team will conduct in-depth interviews with up to 12 YMSM and 12 YTW and GNC youth, up to 10 focus groups with a maximum of 40 YMSM and 40 YTW and GNC youth, and up to 40 brief interviews with LGBTQ-inclusive employers. Participants will be recruited through a community-based recruiter, passive recruitment in community spaces and on social media, and active recruitment by research staff in community spaces serving LGBTQ youth.

**Results:**

In-depth interviews were conducted with 21 participants, and 7 focus groups were conducted with 46 participants in total. In addition, 19 brief interviews with LGBTQ-inclusive employers were conducted. The analysis of the data is underway.

**Conclusions:**

Preliminary findings from the formative phase of the study will be used to inform the tailoring and refinement of the iFOUR adult-based intervention into the youth-focused W2P intervention curriculum. Perspectives from YMSM, YTW, GNC youth, and LGBTQ-inclusive employers offer a multidimensional view of the barriers and facilitators to adolescent and young adult LGBTQ employment. This information is critical to the development of a culturally appropriate and relevant youth-focused intervention.

**Trial Registration:**

ClinicalTrials.gov NCT03313310; https://clinicaltrials.gov/ct2/show/NCT03313310

**International Registered Report Identifier (IRRID):**

DERR1-10.2196/16384

## Introduction

### Background

In the United States, young cisgender men who have sex with men (YMSM), young trans women (YTW), and gender-nonconforming (GNC) youth who are assigned male at birth experience high rates of HIV infection [1,2]. In 2017, roughly 39,000 people were diagnosed with HIV, with 13- to 24-year-olds representing 21% of all new diagnoses [1]. Among these adolescent and young adult HIV diagnoses, 81% were attributed to male-to-male sexual contact, according to the Centers for Disease Control, and 75% were among black and Latinx youth [1]. Although precise estimates of HIV infection among YTW and GNC youth are sparse, a meta-analysis of 88 studies found that an average of 14% of trans women tested positive for HIV across studies, whereas 16% self-reported a known HIV-positive status [2]. In a separate study of YTW of color, aged 16 to 24 years, Garofalo et al [3] found that 22% of YTW reported being HIV positive, and 59% reported engaging in condomless anal sex in the past year. HIV among YTW, YMSM, and GNC youth is further complicated by high rates of other sexually transmitted infections (STIs), low rates of HIV testing [4,5], high rates of HIV risk behaviors, and poor outcomes at each step of the HIV continuum of care [6-8]. However, for YMSM, YTW, and GNC youth of color, these outcomes are influenced by important social contextual factors, including social isolation, economic marginalization, and unmet HIV-prevention needs [3,9-15]. Addressing the social and structural drivers of HIV risk is critical to decreasing HIV incidence, particularly among YMSM, YTW, and GNC youth of color [16,17]. Phase 2 of this study has also been published [18]. 

### Social and Structural Determinants of HIV Risk

Black YMSM, YTW, and GNC youth between 13 and 24 years accounted for 43% of all new adolescent and young adult HIV diagnoses, whereas Latinx YMSM, YTW, and GNC youth accounted for approximately 21% [1]. Given that census estimates indicate black and Latinx youth of all gender and sexual identities constitute approximately 14% and 23% of the adolescent and young adult population, respectively [19], these young people bear a disproportionate burden of infection. Although individual behaviors play a part in HIV acquisition, these disparities must also be understood in the context of structural factors that place these young people at an increased risk for HIV exposure [3,4,15,20-22]. Black and Latinx YMSM, for instance, have been shown to have lower levels of access than their white peers to pre-exposure prophylaxis (PrEP), an efficacious HIV-prevention medication [23]. Additionally, despite advancements in civil rights for people of color and LGBTQ individuals, LGBTQ people of color continue to face persistent stigma, discrimination, and victimization in school, the workplace, housing, and health care, related to their identities [6,7,24-28].

For YMSM, YTW, and GNC youth of color, experiences of bullying, harassment, and violence may mean that schools are unsafe environments [25,29]. Such social marginalization may force the youth to leave school altogether [25,30,31]. In the 2015 US Transgender Survey, 17% of transgender and GNC respondents indicated that they faced such severe mistreatment that they left a K-12 school, and nearly a quarter of respondents who reported being out or perceived as trans or GNC in college or vocational school had been verbally, physically, or sexually assaulted [26]. Among black respondents, outcomes were worse. Approximately 22% of black respondents had left a K-12 school due to mistreatment, and 10% had been expelled. Black YTW indicated a higher likelihood of having been verbally harassed (67%), physically attacked (55%), and sexually assaulted (38%) because of their trans identity compared with respondents of other genders. The school experiences indicated by YMSM, YTW, GNC youth of color lead to lower educational attainment and place marginalized youths at increased risk for subsequent employment challenges, associated economic instability, and negative health outcomes.

Sexual and gender minority youth of color also contend with additional challenges when seeking employment, including hiring bias, job discrimination, unequal pay, limited benefits, and having less access to tailored career services than their white peers [32,33]. Furthermore, men who have sex with men (MSM), trans women, and GNC individuals of color report having to navigate unwarranted background checks, limited support in the workplace, and an absence of nondiscrimination laws to protect them from discrimination [32]. As a result, a substantial proportion of YMSM, YTW, and GNC youth of color live in poverty and experience negative structural outcomes, including homelessness, unemployment, and limited access to health care, LGBTQ services, and HIV prevention and care [6,10-14,34].

Faced with limited economic options and protections and often lacking traditional familial support [35,36], some black and Latinx YMSM, YTW, and GNC youth migrate to nontraditional economies as a means of survival. This work may include various forms of cash-for-service work, including unregulated labor, such as cleaning, childcare, or the exchange of sex for money, drugs, or food. A study of trans people living in Washington, DC, found that 76% (n=151) of YTW aged 15 to 24 years reported engaging in sex work, with 35% having done so in the past 3 months [9]. Among HIV-positive YTW, 23% were involved in sex work, compared with 6% who were not [9]. In a large study of YMSM of color (n=3316; median age 19 years), roughly 12% reported engaging in sex work at some point in the past 6 months [37]. Given the illegal nature of this work, migration to these nontraditional economies places black and Latinx YMSM, YTW, and GNC youth at risk for further marginalization and economic instability, should they be arrested. In addition, sex work can place these young people at increased risk for HIV and STIs through an increased number of sexual partners, exposure to higher prevalence sexual networks, and power dynamics that present barriers to negotiating condom use [38-43].

### The Role of Structural Interventions in Reducing HIV Risk

Structural interventions facilitate the development of agency by intervening in systems and structures that constrain choice and have the potential to promote the uptake of behavioral and biomedical approaches to risk reduction [16,17]. For example, structural interventions, such as comprehensive sex education and increased health care coverage, have significantly reduced the incidence of HIV [44-50]. Although the impact of structural interventions on HIV incidence may not be seen immediately, these interventions can effect changes in a community’s cultural, legal, political, and economic context that may facilitate subsequent reductions in HIV vulnerability [16,17]. Scalable but potentially high impact, structural interventions that trace and disrupt the pathways between social and economic marginalization and adolescent and young adult HIV infection are sorely needed [16,17,34]. However, few structural interventions targeting adolescent and young adult sexual and gender minorities exist, and we know of no studies that have explicitly examined the role of employment on HIV risk and prevention for black and Latinx YMSM, YTW, and GNC youth.

This paper aimed to describe the protocol for the exploratory phase of Work2Prevent (W2P). The purpose of this formative phase of the study is to determine the essential components of an employment intervention aimed at increasing job-seeking self-efficacy and career readiness among black and Latinx YMSM, YTW, and GNC youth aged 16 to 24 years vulnerable to HIV infection. This includes identifying target population needs, barriers, strengths, preferences, and norms. The findings from this phase will be used to adapt and refine increased individual income and independence (iFOUR), an effective employment workshop series for HIV-positive adults [51], to the needs of YMSM, YTW, and GNC youth of color in Chicago, Illinois.

## Methods

### Study Design

This exploratory phase of the W2P study consists of in-depth interviews and focus groups with members of the target community as well as brief interviews with LGBTQ-inclusive employers, which will be used to adapt and refine iFOUR [51]. Given the increasing expansiveness of identity labels and expression, the study eligibility criteria pertaining to gender are intentionally broad, including individuals assigned male at birth with a range of gender identities. The study will take place in Chicago, which is the intended site for piloting the resulting intervention.

In-depth, semistructured interviews with YMSM, YTW, and GNC youth will be conducted to assess the individual-, social-, and structural-level factors that influence participants’ employment and employment-seeking experiences, career trajectories, and career-related aspirations. Semistructured interviews ranging from 1 to 2 hours will be conducted by trained staff. Sample interview questions include (1) *Please walk me through all of the jobs you’ve had, starting with your very first paid employment*; (2) *How do you find out about jobs you might want to apply for?* (3) *What kinds of challenges have you faced when starting a new job as a young gay or bisexual man OR trans woman or gender-non conforming youth of color?* (4) *Imagine you could give a younger version of yourself advice around employment. What advice would you give yourself?* (5) *Can you describe any career-related goals you may have? How are you working toward those goals?* (6) *Has anyone helped you with your employment goals? Who were they, and what kinds of support did they provide?* (7*) How do you get by when money is tight?* (8) *Has anything gotten in the way of applying to jobs in the past?* (9) *How do you handle stress-related work or money?* Participants will also complete a short paper survey to capture sociodemographic and behavioral information.

Focus groups with black and Latinx YMSM, YTW, and GNC youth will assess community norms, attitudes, motivations, and perceptions regarding employment. Focus groups are facilitated by trained staff, include 4 to 8 participants, and will range from 1 to 2 hours. Participants will also complete a short paper survey to capture sociodemographic and behavioral information. Focus groups will be separated by self-identity (groups with YMSM and groups with YTW and GNC youth) so that gender-related differences can be explored. Sample focus group questions include (1) *What is the value of having paid employment? What does having a job mean to you?* (2) *What factors most help gay and bisexual men OR trans women and gender-nonconforming youth get and keep jobs?* (3) *What factors prevent gay and bisexual men OR trans women and gender-nonconforming youth from getting and keeping jobs?* (4) *What is a typical job for a young gay and bisexual man OR young trans woman and gender-nonconforming youth? Why do you think these jobs are so common?* (5) *What makes a work environment LGBTQ-friendly? What could employers do to promote a supportive and safe environment?* (6) *What topics do you think would be important to cover in an employment workshop program for young gay and bisexual men OR trans women and gender-nonconforming youth of color?*

Brief interviews will be conducted with representatives from LGBTQ-inclusive employers to assess employer-level barriers and facilitators to securing and maintaining stable employment for YMSM, YTW, and GNC youth. Short, structured interviews will be implemented by trained staff and range from 10 to 15 min. Participants will also complete a short paper survey to capture information on employer policies and training regarding LGBTQ employees. Sample interview questions include (1) *How do you recruit LGBTQ employees to your organization?* (2) *How do you communicate LGBTQ inclusive policies and benefits to prospective and current employees?* (3) *What barriers to hiring and retaining young gay and bisexual men color, in particular, have you experienced, or have you witnessed?* (4) *What barriers to hiring and retaining young trans women and gender-nonconforming youth of color, in particular, have you experienced or witnessed?* (5) *Can you tell me about a time when an employee at your organization has experienced an incident of antiLGBTQ harassment or conflict? How did you or others at your organization respond?* These data will be analyzed to inform modules in the intervention focused on strategies for selecting and vetting of employers who report being LGBTQ-inclusive.

### Study Setting

Interviews and focus groups with YMSM, YTW, and GNC youth will be conducted in a private room on the University of Chicago campus. Interviews with LGBTQ-inclusive employers will be conducted off-site at the employers’ facilities or community-based LGBTQ job fairs.

### Participants

The study team will conduct in-depth interviews with up to 12 YMSM and 12 YTW and GNC youth and up to 10 focus groups with a maximum of 40 YMSM and 40 YTW and GNC youth in total. Inclusion criteria include (1) being assigned male at birth; (2) identifying as YMSM, YTW, or GNC youth; (3) identifying as African American, black, Hispanic, or Latinx; (4) 16 to 24 years of age; (5) English-speaking; and (6) seeking employment or currently employed.

Additionally, up to 40 interviews with representatives of LGBTQ-inclusive employers will be conducted. Inclusion criteria include (1) being an employer or manager at an LGBTQ-inclusive company, defined as a company with a rating of 80% or higher on the Human Rights Campaign Foundation’s Corporate Equality Index, a company currently listed as an Out & Equal, LGBT CareerLink employer, a nonprofit organization with an LGBTQ-focused mission, an LGBTQ-owned small business with <200 employees, or an organization participating in an LGBTQ job fair; (2) being aged 18 years or older; (3) speaking English as a primary language; and (4) located in the Chicago area.

### Recruitment

YMSM, YTW, and GNC youth will be recruited to participate in interviews and focus groups by the research staff and study recruiters who are also members of the target population. Focus group and key informant interview participants will be recruited by the study staff through pre-existing network connections, including the Chicago Center for HIV Elimination, and snowball sampling.

LGBTQ-inclusive employers will be recruited actively by the research staff. Planned recruitment strategies include attending LGBTQ job fairs and emailing LGBTQ nonprofits, LGBTQ-owned small businesses, and employers in the Human Rights Campaign Foundation’s Corporate Equality Index or Out & Equal, LGBT CareerLink lists.

### Compensation

YMSM, YTW, and GNC youth will receive US $40 for participating in the in-depth interviews and focus groups. LGBTQ-friendly employers will be offered a US $10 gift card for completing the brief interview.

### Data Collection

All interviews and focus groups will be recorded and transcribed. Transcripts will be subsequently verified and deidentified by the research team. All audio recordings will be destroyed on completion of the study. Surveys are completed on paper and entered into a computer-based data entry system by trained research staff. All data entry will be reviewed by the research team for quality assurance. All study records, including audio recordings, transcripts, and surveys, will be labeled with a unique participant identification number rather than identifying information. Paper records will be securely stored in locked filing cabinets at the University of Chicago. Electronic records will be securely stored in the University of Chicago network and the Sharepoint site for Adolescent Medicine Trials Network for HIV/AIDS Interventions (ATN) at the University of North Carolina at Chapel Hill.

### Analyses

The analytical plan includes a thematic content analysis of the interviews and focus groups and a descriptive statistical analysis of the survey data. The analysis of the interviews and focus groups will proceed in 2 steps. First, open-coding will be conducted by trained qualitative researchers to identify preliminary emergent themes. Next, this open-coding will be used to develop a codebook for the second stage of analysis. For a subsequent qualitative analysis, 2 independent coders will each use the developed codebook to analyze the data, and the coding schema will be compared for interrater reliability using the Cohen kappa coefficient. Given that the data focus on the experiences of individuals at the intersection of multiple marginalized identities, members of the analytic team will consider the roles of their own social locations and the social locations of the YMSM, YTW, and GNC youth of color engaged and discussed in the data [28,52,53]. In addition, the data will be interpreted with respect to the contexts in which these young people live and work, for example, racial, cultural, and socioeconomic contexts [54]. Descriptive statistics will be used to analyze the proportions and central tendencies for the survey data. All qualitative coding will occur in Atlas.ti 7 (Scientific Software Development) [55], and statistical analysis will occur in SPSS 25 (IBM Corporation) [56].

### Refinement and Adaptation of the Intervention

Preliminary analysis of the qualitative data will be used to inform the adaptation and refinement of iFOUR, an existing effective employment intervention curriculum for HIV-positive adults [56]. Curriculum modules will be revised or developed to (1) address the specific needs and barriers highlighted by the youth in the qualitative data; (2) leverage the strengths highlighted by the youth in the qualitative data; (3) include examples that are culturally and developmentally aligned with the experiences described by the youth in the qualitative data; (4) incorporate topics that the youth suggested in the qualitative data; (5) assist the youth in identifying LGBTQ-inclusive employers and navigating their hiring processes based on the insights from the employer interviews. A Youth Advisory Board will also review all existing, revised, and newly developed curriculum material for further tailoring and feedback.

### Ethics, Consent, and Institutional Board Approval

The research and procedures presented in this study protocol have been approved by the University of Chicago and the University of North Carolina at Chapel Hill Institutional Review Boards (IRB), IRB 16-1152 and 17-0795, respectively. The National Institute of Child Health and Human Development provided a Certificate of Confidentiality for the study. A waiver of parental consent was obtained for participants aged 16 to 17 years. The study is registered on ClinicalTrials.gov (NCT03313310).

All participants will complete a signed written consent form before participation in interviews or focus groups. This form details information on study procedures, risks, and benefits of participation. As part of the informed consent process, research staff explain the study and consent form to each participant. To ensure comprehension, staff ask participants to summarize the study in their own words before asking them to sign the form. Consent forms are stored securely and will be kept for up to seven years, per our IRB protocol.

## Results

In-depth interviews have been conducted with 21 participants between May and November 2017. Seven focus groups were conducted with 46 participants between May and September 2017. In addition, 19 brief interviews with LGBTQ-inclusive employers have been completed in September 2017. A qualitative analysis of the data is underway. Preliminary findings from these data were used to inform the development and refinement of the W2P intervention curriculum. The development of the intervention will be described in a separate manuscript.

## Discussion

W2P is a structural-level employment intervention aimed at increasing job-seeking self-efficacy and career readiness among black and Latinx YMSM, YTW, and GNC youth, aged 16 to 24 years. This formative phase is a critical first step in assessing the structural needs of youth populations disproportionately impacted by HIV to optimally tailor structural-level interventions. Although individual-level interventions that target behaviors such as condom and PrEP use play a significant role in the reduction of HIV transmission, such interventions are enacted within a context influenced by social and structural factors. Given the range of barriers that many marginalized individuals experience, intervention at the structural-level can facilitate increased agency over a person’s sexual health and associated decision making. W2P seeks to identify and remove barriers to black and Latinx YMSM, YTW, and GNC youth enacting change toward their own success and economic independence and stability.

Data collected in this phase of W2P are critical to adapting the adult-based iFOUR curriculum [49] to black and Latinx YMSM, YTW, and GNC youth. This initial phase of W2P provides clarity on the needs of the population to increase the cultural and developmental appropriateness of the intervention curriculum. Furthermore, interviews and focus groups will allow the team to identify experiences shared across the population to ensure that intervention material, including exercises and examples, are relevant. Participants will also be encouraged to communicate their preferences for an employment intervention if they were to take part in one. Although the formative phase of this project serves an important role, it may not capture nor be able to incorporate all relevant factors into one standalone intervention. Nevertheless, it may generate data that can be used to inform future interventions along with the W2P curriculum. W2P will target key facilitators of job readiness and job-seeking self-efficacy and ultimately assist the youth in securing and sustaining employment. Although identifying support networks to achieve goals is included in the curriculum, the intervention will not necessarily directly engage individuals’ support systems. Thus, subsequent social and emotional support informed by data from this exploratory phase of the study may be necessary to augment the core components of the intervention.

The formative work described in this protocol may not be generalizable to the broader LGBTQ youth population. In particular, employment challenges faced by black and Latinx YMSM, YTW, and GNC youth in Chicago may not be representative of nonurban areas. Rural and periurban areas may face geographic challenges, including job scarcity, outsourcing, or erasure of industries, as well as agricultural and seasonal employment, which are beyond individual-level control and ultimately create a different employment landscape compared with the youth in urban settings. Nevertheless, the goal of the intervention design is to address the most pervasive challenges YMSM, YTW, and GNC youth of color face when seeking employment. Additionally, the formative phase of this study may underscore the necessity of additional resources beyond the scope of the intervention. Increased educational attainment such as completing high school or a General Educational Development program, housing stability, and criminal record expungement is strongly linked to employment outcomes but may not be directly addressed in the intervention curriculum. Therefore, providing a network of referral systems to additional social services and support may be a necessary part of the intervention.

In summary, phase 1 of the development of the intervention will provide the necessary inputs to create W2P, a youth-centered and relevant job readiness and employment intervention. Economic stability and employment play a critical role in mitigating the migration of the youth to nontraditional and unregulated labor economies that are associated with HIV risk and exposure, such as survival sex work. Equipping the youth with the skills and resources to seek and attain employment offers an opportunity to address the social determinants of adolescent and young adult HIV, without explicitly focusing on individual-level sexual risk behaviors. Empowerment models that highlight the social capital and assets of the youth are essential in creating culturally and developmentally appropriate and relevant interventions for vulnerable youth, ultimately increasing uptake and efficacy.

## Abbreviations

ATN: Adolescent Medicine Trials Network for HIV/AIDS Intervention
GNC: gender nonconforming
iFOUR: increased individual income and independence
IRB: Institutional Review Board
LGBTQ: lesbian, gay, bisexual, transgender, and queer
MSM: men who have sex with men
PrEP: pre-exposure prophylaxis
STI: sexually transmitted infection
W2P: Work2Prevent
YMSM: young men who have sex with men
YTW: young transgender women
